# Complete Genome Sequence of *Mycoplasma hominis* Strain Sprott (ATCC 33131), Isolated from a Patient with Nongonococcal Urethritis

**DOI:** 10.1128/genomeA.00771-15

**Published:** 2015-07-09

**Authors:** Michael J. Calcutt, Mark F. Foecking

**Affiliations:** Department of Veterinary Pathobiology, University of Missouri, Columbia, Missouri, USA

## Abstract

Presented here is the complete and annotated genome sequence of *Mycoplasma hominis* Sprott (ATCC 33131). The chromosome comprises 695,214 bp, which is approximately 30 kb larger than the syntenic genome of *M. hominis* PG21^T^. Tetracycline resistance of strain Sprott is most probably conferred by the *tetM* determinant, harbored on a mosaic transposon-like structure.

## GENOME ANNOUNCEMENT

*Mycoplasma hominis* was the first human mycoplasma species to be isolated (1), and in 16S rRNA-based phylogenetic schemes, it lends its name to the hominis cluster of taxa (2). Several representatives have been sequenced (3–5), but prior to the commencement of this work, no tetracycline-resistant isolates had been decoded. Accordingly, the genome of *M. hominis* Sprott, a tetracycline-resistant isolate from the urethral discharge of a nongonococcal urethritis patient (6), was determined. While this project was under way, the genome of tetracycline-resistant strain PL5 was reported (5).

*M. hominis* Sprott (ATCC 33131) was sequenced using single-molecule real-time sequencing at the National Center for Genome Resources, Santa Fe, NM. Following assembly (with HGAP version 2 [7]), the 695,214-bp genome (27.4% G+C content, 360× coverage) was annotated using Prokka (8) to identify RNA features and coding sequences (CDSs) and then refined by comparison to the manually curated genome of *M. hominis* LBD-4 (4). Among the 615 genes, 549 CDSs, 6 rRNAs, and 33 tRNAs were identified. The sequences of 24 pseudogenes were confirmed by an inspection of aligned reads or by PCR/Sanger sequencing.

Consistent with what has been reported for pairwise alignments of other *M. hominis* genomes (4, 5), no large-scale rearrangements were evident. Strain Sprott did not contain the MHoV-1 prophage genome (4) but harbored a 25,256-bp mosaic *tetM*-containing transposon analogous to that recently identified in PL5 (5). A search for additional mobile elements revealed pseudogenes for transposases and two truncated genes with greatest similarity to open reading frames (ORFs) contained in mycoplasmal integrative conjugative element (ICE) units (9, 10).

In addition to the reported OppA and Vaa adhesins (11, 12), an analysis of predicted surface proteins identified tandem paralogs of the thiamine pyrophosphate (TPP)-binding lipoprotein p37 (13), with a TPP riboswitch (14) within the intergenic region and two putative polyamine-binding proteins linked to a cognate ABC transporter. These paralogs may exhibit redundant functions or represent degrees of binding discrimination for related polyamines, as noted for *Escherichia coli* PotD and PotF (15). Further redundancy or overlapping specificity may also exist for three lipoproteins that are significant matches to the polynucleotide-binding lipoprotein of *Mycoplasma gallisepticum* (16).

The deduced metabolic pathways for *M. hominis* were comprehensively reviewed in the report accompanying the PG21 genome release (3). One additional component was recently identified through an analysis of an accessory/scavenging pathway for fatty acid biosynthesis in *Staphylococcus aureus* (17), with implications for *Mycoplasma* species that lack *de novo* pathways. The authors noted an FakA (fatty acid kinase subunit A)-encoding homolog in an equivalent genomic locale in *Mycoplasma pneumoniae*. Interrogation of the *M. hominis* genome also revealed an orthologue, indicating a broader distribution among additional *Mollicutes* taxa. Although FakA contains a DegV (fatty acid-binding) domain, one of two additional DegV domain-containing subunits is required for fatty acid kinase activity in *S. aureus*. MHOMSp_01495 is the sole match to either *S. aureus* FakB paralog.

This data set further informs our understanding of genetic variation, aspects of metabolism, and the role of lateral gene transfer in the acquisition of antibiotic resistance for this important human mycoplasmal species.

### Nucleotide sequence accession number. 

This complete genome sequence has been deposited at DDBJ/EMBL/GenBank under the accession no. CP011538.
